# Testing the Moderating Effect of Anti-Prejudice Motivation and Peer Attitudes on the Effectiveness of a School-Based Vicarious Contact Intervention

**DOI:** 10.1007/s10964-024-01985-w

**Published:** 2024-04-18

**Authors:** Viivi Mäkinen, Inga Jasinskaja-Lahti, Tuuli Anna Renvik, Karmela Liebkind

**Affiliations:** University of Helsinki, Helsinki, Finland

**Keywords:** Vicarious contact, Prejudice-reduction, Anti-prejudice motivation, School intervention, Peer attitudes

## Abstract

Vicarious contact has often been used for studying prejudice-reduction in school contexts due to its relatively accessible application through written or audiovisual portrayals of positive intergroup contact. However, these interventions may sometimes prove ineffective, thus restricting their ecological validity and independent use in education. To contribute to the understanding of factors that might facilitate or mitigate the efficacy of vicarious contact in reducing ethnic prejudice among adolescents, the present study tested for the moderating effect of anti-prejudice motivation and friends’ outgroup attitudes. Participants were Finnish secondary school students (*N* = 334; *M* = 13.38 years, SD = 0.53; 48% female; 19% ethnic minority) allocated into cluster-randomized intervention (*N* = 149) and control (*N* = 185) groups. Participants in the intervention group took part in 4 × 45-min teacher-led intervention sessions. A pretest-posttest design was employed to assess the outgroup attitudes three weeks before the intervention and the follow-up two weeks after. The results showed that adolescents’ intrinsic, but not extrinsic, anti-prejudice motivation and the pre-intervention attitudes of their reciprocal classroom friends positively predicted post-intervention attitudes towards people from different ethnic and cultural groups. However, only extrinsic motivation moderated the intervention effect as the results indicated the intervention to have a detrimental effect on outgroup attitudes among adolescents with less motivation to be non-prejudiced in order to gain social acceptance. This attitudinal backlash among adolescents less susceptible to the social influence of others implies that motivational aspects should not be overlooked when developing school-based intervention programs, especially when social norms are used as a mechanism of attitude change.

## Introduction

Previous research has offered empirical evidence for the effectiveness of direct and indirect forms of intergroup contact in improving outgroup attitudes among children and adolescents (Ülger et al., 2018). However, less is still known about the factors determining for whom and under which conditions these efforts to reduce ethnic prejudice are the most effective—or ineffective. One relatively neglected aspect in this regard is the role of normative social influence, although the need to gain social acceptance by complying with peers is especially present in adolescence (e.g., Crone & Dahl, 2012). To fill this gap in the literature, this study revisits data from an earlier school-based vicarious contact intervention that did not produce a direct intervention effect (Mäkinen et al., 2022) and tests the moderating role of classmates’ attitudes and participants’ socially/internally derived anti-prejudice motivation in improving attitudes towards ethnic and cultural outgroups among secondary school students in Finland, a Nordic country with a relatively small population of people with an immigration background.

### Vicarious Intergroup Contact

Vicarious contact, originating from vicarious learning, is a form of indirect intergroup contact that extends the idea of the contact hypothesis (Allport, 1954) with the principles embodied in social cognitive theory (Bandura, 1986). In accordance with the social cognitive theory that emphasizes how attitudes and behaviors are socially learned by observing relevant others, vicarious contact relies on role models who engage in contact with an outgroup member on behalf of the observer (Mazziotta et al., 2011). This sets an example of positive intergroup interaction, contributing to the development of positive attitudes towards the outgroup. Since the formulation of the concept (Mazziotta et al., 2011; see also Wright et al., 1997 for extended contact), empirical evidence on the prejudice-reducing effect of vicarious contact has emerged (for a review, see Vezzali et al., 2014). Previous studies have also identified several mediators explaining both the affective (e.g., decreased anxiety and intergroup threats) and cognitive (e.g., in- and outgroup norms and perspective-taking) route of indirect contact effects (see, Vezzali et al., 2014), thus adding to the understanding of the mechanisms behind vicarious contact.

Due to its relatively accessible application, usually through written stories or films portraying positive cross-group contact, vicarious contact has often been utilized in a school context to improve children’s and adolescents’ attitudes toward people from different stigmatized outgroups (Di Bernardo et al., 2017). However, despite this extensive use in promoting harmonious intergroup relations in real-life settings, vicarious contact interventions might not always yield the desired results. For instance, vicarious contact might sometimes work only for a certain sub-group of participants, such as girls, but not boys, with more negative outgroup attitudes at the outset (Liebkind et al., 2019), or even have a counterproductive effect on others, for example in the form of increased feelings of intergroup anxiety among older adolescents (Liebkind et al., 2014). To provide schools with ecologically valid means for supporting the development of harmonious intergroup relations, it is thus vital to understand the factors that can help or hinder the effective use of vicarious contact.

Social cognitive theory (Bandura, 1986) posits that in addition to observing the role models’ behavior, individuals also evaluate its social consequences, thus increasing or decreasing the likelihood of the behavior being repeated. This implies that what matters is not only the example set by the observed role models but also the normative support for their actions as expressed by others in the immediate social surroundings (Bandura, 2002). In line with developmental intergroup theory (Bigler & Liben, 2007), many socio-normative factors can impact the extent to which a prejudice-reduction intervention succeeds in its aims, and these factors can also be intertwined with the developmental changes in specific age groups. For instance, the influence of family and classroom context is central to the socialization of ethnic prejudice in youth (Bobba et al., 2024), which is also a period for maturing social motives and the adoption of social and societal norms (Crone & Fuligni, 2020). This can make adolescents an especially potential age cohort to benefit from vicarious contact, which relies on social learning and modeling others.

Despite the centrality of social influence in attitude formation in adolescence, previous studies have not fully considered the role of others’ perceived approval in contributing to the effectiveness of vicarious contact conducted among youth. Rare exceptions include studies that have highlighted the importance of perceived support for vicarious intergroup contact expressed directly or indirectly by peers (Cocco et al., 2022) or different authority figures, such as coaches (Gómez & Huici, 2008) and teachers facilitating a classroom intervention (Mäkinen et al., 2022). For example, the perceived engagement of the intervention facilitators in conducting a school-based vicarious contact intervention has been shown to facilitate the positive effect of the intervention on participants’ outgroup attitudes (Mäkinen et al., 2022). Similarly, the attitudes of the closest classmates taking part in the same intervention could act as a crucial catalyst for attitude change, influencing how successful an intervention is in reducing prejudice. In addition, adolescents can differ regarding the extent to which their motivation to be non-prejudiced relies on such social cues or more on internalized egalitarian beliefs (e.g., Thijs et al., 2016), which might again affect the intervention’s effectiveness.

### Influence of Friends and Peers on Outgroup Attitudes

Research on child development has shown friendships to have beneficial effects on the psychosocial development of children and adolescents, contributing positively to their emotional, cognitive, and behavioral functioning (see, e.g., Vitaro et al., 2009). Similarly, friends and peers are also important for the development of outgroup attitudes (Pehar et al., 2020). This is especially true in adolescence, which is considered a crucial developmental phase for attitude formation (Dunham & Degner, 2010) and marks a period when people are sensitive to influences from their social surroundings (Crone & Dahl, 2012). In contrast to childhood years, prejudice towards outgroup members thus seems to become more context-dependent during the transition to adolescence (Raabe & Beelmann, 2011). However, studies also suggest that this susceptibility to peers´ attitudes is primarily characteristic of early adolescence and diminishes later with increasing maturity (Ahmed et al., 2020). According to previous studies, this decreasing reliance on peer influence would take place between the ages of 14 and 18 (Steinberg & Monahan, 2007).

The focal role of peer norms in forming outgroup attitudes in early adolescence suggests that peers taking part in the same classroom intervention can be salient agents of social influence. However, only a few previous studies have examined the role of classroom friends in the effectiveness of a contact-based prejudice-reduction intervention. For instance, in an intervention study using vicarious contact to target stigma-based bullying among Italian primary school children (Cocco et al., 2022), the participants were assigned either to work individually or collectively in activities designed to reinforce the message of the intervention. The results showed that vicarious contact was associated with more prominent intentions to react to bullying toward children of foreign origin when participants had collectively negotiated responses to reinforcing activities. Similar findings have also been obtained with imagined contact interventions. In a study testing the effectiveness of norm-framed imagined contact among primary school-aged children in Ireland, asking participants to imagine meeting a refugee child with their classmates in the classroom context was associated with less affective and cognitive bias towards refugees and less bias regarding contact intentions as compared to dyadic imagined contact (Smith & Minescu, 2022).

However, these few prior studies on the impact of classroom peers on the effectiveness of indirect contact interventions in schools have focused on the presence of peers as an experimental condition instead of investigating the impact of classmates’ self-reported attitudes per se. As an exception, a study conducted among Hungarian high school students tested the influence of friends’ attitudes on the effectiveness of direct intergroup contact on youth’s attitudes towards Roma and LGBT minorities (Orosz et al., 2016). The results showed that peers’ prejudiced attitudes did not impact the intervention’s effectiveness. Instead, the direct contact with members of the Roma and LGBT minorities through the Living Library program reduced prejudice toward these groups among all participants regardless of their friends’ attitudes. It is, however, crucial to note that affecting outgroup attitudes through ingroup norms is a mechanism that is more characteristic of indirect than direct contact (De Tezanos‐Pinto et al., 2010). Thus, friends’ positive attitudes might have a more substantial buffering effect on different forms of indirect contact, such as vicarious contact.

### Anti-Prejudice Motivation

Although social norms inform us about the social acceptability of prejudice, why and when those norms are followed can be up to an individual’s motivation to regulate prejudice (e.g., Plant et al., 2003). Individuals can be motivated to be non-prejudiced because they have internalized egalitarian beliefs and values that they find personally meaningful, while for others, the motivation to be non-prejudiced can rely primarily on external pressure to adhere to prevailing anti-prejudice norms to avoid others’ disapproval (Plant & Devine, 1998). Despite this distinction between intrinsic and extrinsic anti-prejudice motivation being often described in a dichotomous manner, the two motivational facets for regulating prejudice are independent, meaning that the motivation for responding without prejudice can be either extrinsic or intrinsic, both or neither. In fact, for most people, the motivation to respond without prejudice emanates from personal dedication to egalitarian values and perceived social pressure (Bamberg & Verkuyten, 2022).

From a developmental perspective, both intrinsic and extrinsic motivation to be non-prejudiced begin to emerge by late childhood, which might stem from several social, moral, and cognitive developments around that developmental stage (Hughes et al., 2016). For example, showing more moral concerns for equality and the welfare of others (Rizzo et al., 2016), but also increased awareness of being evaluated based on the adherence to in-group norms (Abrams et al., 2007) might explain the increase of both intrinsic and extrinsic anti-prejudice motivation in children. Similarly to studies conducted among adults (see, e.g., Legault et al., 2007), intrinsic and extrinsic anti-prejudice motivations in late childhood and adolescence have often been shown to predict different attitudinal and behavioral outcomes in intergroup relations. For instance, intrinsic anti-prejudice motivation has been associated with more positive outgroup attitudes (Jargon & Thijs, 2021) and less ethnic bias (Thijs et al., 2016), while extrinsic anti-prejudice motivation generally predicts more negative attitudes toward members of ethnic outgroups (Jargon & Thijs, 2021) and is related to more intergroup anxiety (Hughes et al., 2016).

Prior research on anti-prejudice motivation has demonstrated that certain normative cues may be more effective than others in tackling prejudice if they stimulate intrinsic rather than extrinsic anti-prejudice motivation. For instance, in a cross-sectional study among pre-adolescents, the link between norm perception and outgroup attitudes via intrinsic anti-prejudice motivation was shown to be more prominent for the normative message underlining equality than for the ones highlighting the moral rules of being nice and honest and not being mean to people from other countries and cultures (Jargon & Thijs, 2021). Given their rule-like nature, these latter messages convey more external control to regulate prejudice, which, in some studies, has also been shown to worsen attitudes. For instance, in an experimental study among university undergraduates, motivating non-Black participants to reduce racial prejudice by emphasizing external control produced more explicit and implicit prejudice than not intervening at all (Legault et al., 2011). Similarly, previous studies have found that perceived pressure to adhere to other-imposed non-discriminative norms can even prompt negative affective responses, leading to an attitudinal and behavioral “backlash” among strongly externally motivated people (Plant & Devine, 2001).

Prior empirical findings thus stress the beneficial effect of fostering intrinsic anti-prejudice motivation over extrinsic motivation, which is more likely to have a detrimental effect on outgroup attitudes. However, it is crucial to note that even strongly extrinsically motivated individuals, unlike fully a motivated ones (cf., Deci & Ryan, 2002), are also motivated to respond without prejudice but do so for different reasons than people with more intrinsically derived motivation (Legault et al., 2007). These differences in the source of anti-prejudice motivation mean that individuals can vary regarding the extent to which they are sensitive to and concerned with normative pressure to avoid expressing prejudice. For example, a study examining the externally and internally motivated confrontations of intergroup bias on the further expression of stereotyping showed that being confronted about the use of stereotypes during an experimental task in a manner that emphasized normative consequences of the behavior reduced stereotyping among participants scoring low on internal motivation while participants highly internally motivated to avoid bias reduced stereotyping most with an internally framed confrontation (Burns & Monteith, 2019). Hence, by paying more attention to prevailing norms, extrinsically motivated individuals could be more receptive to vicarious contact and more inclined to follow the normative example of positive intergroup contact exposed through observing others’ behavior. Nevertheless, previous research has not examined the role of motivational aspects in the effectiveness of vicarious contact.

### Context of the Study

The study was conducted in Finland, a country in Northern Europe that belongs to the geographical and cultural region of Nordic countries. From a historical perspective, Finland has become a country of immigration moderately late, with more substantial changes in the migration flow taking place only from the 1990s onward. The country remains relatively culturally homogenous: only a small proportion of roughly 5,5 million inhabitants in Finland are foreign-language speakers (8.9%) or belong to a religion other than Evangelical Lutheran (2.8%; Statistics Finland, 2023). At the time of the data collection, of all the children under the age of majority, 8.6 percent had a foreign background either by birth or through their foreign-born parents (Statistics Finland, 2023). The most common countries of origin were Russia or the former Soviet Union (17%), Estonia (13%), Somalia (10%), and Iraq (8%). Other prevalent countries of origin included former Yugoslavia, Syria, Afghanistan, Turkey, Vietnam, China, and Sweden.

## Current Study

Despite the accentuated role of social norms and modeling in producing the vicarious contact effect, previous research on school-based vicarious contact interventions has not fully considered the role of peer influence in contributing to the effectiveness of the intervention among adolescents. This study examines the normative and socio-motivational aspects of reducing prejudice through vicarious contact by testing the moderating role of friends’ attitudes and participants’ intrinsic and extrinsic anti-prejudice motivation on the intervention effect on adolescents’ attitudes towards people from different ethnic and cultural groups. Regarding the friends’ attitudes, the results are anticipated to indicate that the intervention has a more positive effect on outgroup attitudes when friends’ attitudes are positive rather than negative (Hypothesis 1). Regarding adolescents’ motivation to be non-prejudiced, it is anticipated that both high intrinsic and high extrinsic anti-prejudice motivation facilitate the effectiveness of the intervention. Following previous research linking intrinsic anti-prejudice motivation to more positive outgroup attitudes, it is hypothesized that the intervention positively affects outgroup attitudes when intrinsic motivation to be non-prejudiced is high rather than low (Hypothesis 2). On the other hand, relying on the previous research showing externally framed social influence to impact highly extrinsically motivated individuals, it is also proposed that the exposure to positive norms about contact with outgroup members through vicarious contact can result in improved outgroup attitudes among those with socially derived motivation to avoid prejudice. Thus, the intervention is anticipated to have a more positive effect on outgroup attitudes when extrinsic motivation to be non-prejudice is high rather than low (Hypothesis 3).

## Methods

### Participants

The data represents a Finnish sub-sample of a larger data set utilized earlier in (Mäkinen et al., 2022). Participants were students in the 7th (aged 13–14) and 8th (aged 14–15) grades of Finnish lower secondary education. Of the total of 772 students invited to take part in the study, 196 either declined to participate (*n* = 157) or did not participate in the baseline assessments (*n* = 39), thus making the response rate 75% at T1. In addition, 120 participants were lost for attrition (21%) between T1 and T2. Thus, the collected sample consisted of 456 students. Of those, 122 participants were further excluded from the analysis due to the absence of nominated classroom friends (participant not naming any friends, *n* = 79; nominated friends not participating in the study, *n* = 13; none of the nominated friends were reciprocal, *n* = 30) needed for creating a measure for friends’ self-reported attitudes.

The final analyzed sample consisted of 334 students (*M* = 13.38 years, SD = 0.53; 48% female) allocated into intervention (*N* = 149, *M* = 13.44 years, SD = 0.52; 48% female) and control (*N* = 185, *M* = 13.33 years, SD = 0.52; 49% female) groups. The sample consisted mainly of participants belonging to the national majority group (*N* = 270; *M* = 13.38 years, SD = 0.51; 52% boys), while 19 percent of the participants were students with an immigration background, i.e., having at least one foreign-born parent (*N* = *64; M* = 13.34 years, SD = 0.60; 48% female). Most of these students, hereafter referred to as minority participants, were born in Finland (72%). About one-third (39%) had further specified their parents’ country of birth. Reflecting the population structure of immigrants in Finland, the foreign-born parents were mainly from Russia, Estonia, Somalia, and Iraq.

### Procedure

Schools in the capital area of Finland were contacted during the autumn of 2016 and offered an opportunity to participate in the study. Altogether, seven schools were able and willing to accommodate the intervention program into their curriculum in the academic year 2017–2018. Classes within the schools were randomly allocated to experimental and control conditions. The control group followed a regular curriculum, while the experimental group took part in the intervention consisting of four 45-min sessions implemented once a week for four consecutive weeks. The intervention sessions were carried out by the schools’ teaching staff, either teachers or study counselors. The teachers conducted the sessions according to a written facilitator’s manual and were offered a short in-person training by the first author.

The intervention was assessed through a pretest-posttest design: the baseline assessment was conducted three weeks before the intervention and the follow-up two weeks after. In the experimental group, additional assessments asking for students’ evaluation of the intervention were also conducted at the end of the last intervention session. Approval for conducting the study was requested from the school boards of the municipalities, and the intervention sessions were carried out as a part of the curriculum with the permission of the school principals in each participating school. Parental consent for students’ participation in the survey assessments was obtained before the study, following the national ethical guidelines and regulations for research participants under 15 years old (Finnish National Board on Research Integrity, 2019).

### Intervention Program

The “Stories about Friendship” intervention program (Solares et al., 2012) was adapted for use in the present study. During the intervention sessions, the students were presented with written friendship stories printed out for them, reflected on a screen, and read aloud in the class by the students. The main characters narrating the stories represented peer models of the same age, telling how they had met and became friends with a peer from a different ethnic or cultural group (see Appendix for an example of the stories used). To enhance participants’ identification with the narrators, the stories included a short description of the narrator and a picture obtained from a photo-stock. Also, acknowledging the cultural diversity in the participating classes, the narrators in the stories belonged to the Finnish national majority group (four stories) and different immigrant groups (two stories) to avoid portraying only the ethnic majority characters as the active party of intergroup encounters. The ethnic minority narrators and the outgroup friends in the stories told by the majority group narrators represented some of the most prominent immigrant groups in Finland (i.e., people with Russian, Estonian, Somalian, Afghan, Vietnamese, and Arabic backgrounds).

In addition to the written friendship stories, the sessions also included other pedagogical components used for reinforcing the message of the friendship stories and promoting students’ engagement. These included activities such as class discussions led by the teacher and filming short videos in small groups. In these video blogs, students were instructed to narrate their examples of positive intergroup encounters similarly to the friendship stories presented during the intervention sessions. The videos were then shown in the class to utilize the possibility of students acting as positive norm agents for each other. (For a more detailed description of the intervention and study procedure, see Mäkinen et al., 2022).

### Measures

#### Outgroup attitudes

Outgroup attitudes were assessed pre- and post-intervention by asking the participants to indicate their overall feelings towards people from other cultural groups on a commonly used single-item “Feeling Thermometer” (see Lolliot et al., 2015). The 11-point scale ranged from 0° (feelings extremely cold) to 100° (feelings extremely warm).

#### Friends’ self-reported outgroup attitudes

Classroom friends’ self-reported outgroup attitudes were obtained through friend nominations by asking the participants to name their “good friends” from their class at T2. Following the procedure of some previous studies employing friend nominations (e.g., Brenick et al., 2018), students were initially asked to nominate only up to five friends. However, due to the settings of the electronic questionnaires, some participants (*N* = 43) had been able to nominate more than the instructed five classroom friends. As the order of the nominations could not be verified, nominations exceeding five friends were also included. To create a mean score of reciprocal friends’ self-reported attitudes on a scale of 0 to 100, the participants were matched with their friends’ responses on the Feeling Thermometer scale, and the means of reciprocal friends’ T1 and T2 scores were calculated for each participant.

#### Anti-prejudice motivation

Participant’s anti-prejudice motivations were assessed at T1 and T2 with an adapted and shortened version of a measure used earlier with youth samples (Thijs et al., 2016; see also Legault et al., 2007 for the original measure). Before answering the items, the participants were first provided with a short introduction to the concept of prejudice (“If someone is prejudiced, she or he thinks negatively about members of a certain group without really knowing them”) and then asked to respond to a set of items to indicate “Why do you think it is good *not* to be prejudiced?”. The students were introduced to six items, three of them depicting intrinsic motivation, *α* = 0.83 (e.g., “Because I am someone who accepts that people are different from each other”), and the other three depicting extrinsic motivation, *α* = 0.69 (e.g., “Because I don’t want other people to think I’m narrow-minded or dumb”). The items were scored on a 5-point scale ranging from 1 (No, definitely does not apply to me!) to 5 (Yes, definitely applies to me!).

#### Other measures

Along with demographic measures, the students in the experimental condition were asked to evaluate the intervention at the end of the last intervention session. The measure was developed for the descriptive purposes of this study. It consisted of two items: “What did you think of the lessons?” with response options ranging from 0 (really boring) to 100 (really fun), and “How important do you think it is to have such lessons in school?” ranging from 0 (not at all important) to 100 (very important). The two items correlated (*r* = 0.628, *p* < 0.001) and were used to create a composite score for intervention evaluation (Spearman-Brown coefficient *r*SB = 0.772). The mean score of friends’ responses on the two items (*r* = 0.600, *p* < 0.001; *r*SB = 0.750) was composed for each participant to obtain a measure for friends’ evaluation of the intervention.

### Analytical Approach

All statistical analyses were conducted using SPSS IBM version 27 and R software version 4.3.1 (R Core Team, 2023). The item missing values in the analyzed sample were handled with multiple imputations by creating a complete dataset from pooled parameter estimates from twenty imputed datasets. This procedure preserved 11.08 percent of the cases with incomplete data in the variables of interest. Listwise deletion was used to handle the missingness caused by attrition or the lack of data on peer nomination due to participants not nominating any classroom friends or the nominated friends not taking part in the study.

Preliminary descriptive analyses testing for mean differences between condition groups on the demographic and key variables were analyzed using Pearson’s chi-squared test for categorical variables and independent samples t-tests for continuous variables. Regarding the main analyses, the intra-class correlations (ICC), indicating the proportion of variance in the outcome measure at T1, showed no shared variance between schools based on baseline outgroup attitudes (ICC = 0). Also, very little shared variance occurred between classrooms (ICC = 0.043). For this reason, the hierarchical structure of the data was disregarded, and multilevel models were not used. The hypotheses were tested with a 3-step hierarchical regression with outgroup attitudes at T2 as a dependent variable. Participants’ outgroup attitudes at the baseline (T1) and the research condition were entered into the model in Step 1 along with controlling for T1 values of the three moderators (friends’ attitudes, intrinsic motivation, and extrinsic motivation) and participants’ majority or minority group status as a covariate. T2 values of friends’ attitudes and intrinsic and extrinsic anti-prejudice motivation were entered in Step 2, and three interaction terms between condition and the moderators in Step 3. All continuous predictors were z-standardized before being entered into the regression model. Significant interaction was plotted and probed using the regions of significance and confidence bands. The plotting was performed with an online utility (http://www.quantpsy.org; Preacher et al., 2006) and running the generated code in R.

## Results

### Descriptive Analyses

On average, the participants had approximately three reciprocal classroom friends (*M* = 2.70, SD = 1.36). Participants belonging to the majority group mainly had friends who were also members of the national majority group (59%), or the composition of the friend group was cross-cultural (37%). Only a few majority participants had only friends from minority groups (4%). For more than half of the minority participants (63%), all the friends were part of the national majority. About one-fourth (28%) had friends from both majority and minority, while a small number of participants (9%) had only friends who also belonged to a minority group. Regarding the gender of the friends, most participants had only same-sex friends (90%), fewer had both male and female friends (9%), and few had only friends of the opposite sex (1%). Paired samples *t*-tests showed that participants did not differ from their friends regarding their outgroup attitudes at T1 (*t*(333) = −0.70, *p* = 0.482). Also, in the intervention group, participants and their friends did not differ regarding their evaluations of the intervention sessions (*t*(129) = −0.26, *p* = 0.795). In all, the students perceived the intervention relatively favorably, with the mean being above the midpoint of the scale (*M* = 60.07, SD = 20.20).

On average, the participants held more intrinsic (*M* = 4.28, SD = 0.76) than extrinsic (*M* = 3.15, SD = 0.97) motivation to be non-prejudiced (*t*(333) = 18.48, *p* < 0.001). A similar pattern was visible also separately within the intervention (*t*(148) = 13.45, *p* < 0.001) and control (*t*(184) = 12.81, *p* < 0.001) groups. Means, standard deviations, and bivariate correlations for the key variables by condition are shown in Table 1.

**Table 1:** Descriptive statistics and correlations for main study variables by condition

	Intervention (*N* = 149)							Control (*N* = 185)						
Variable	*M*	SD	1	2	3	4	5	*M*	SD	1	2	3	4	5
1. Outgroup attitudes T1	76.31	20.35	–					76.82	21.22	–				
2. Outgroup attitudes T2	75.30	20.88	0.514**	–				77.08	20.85	0.644**	–			
3. Friends’ attitudes T2	77.28	15.25	0.234**	0.279**	–			77.08	17.10	0.270**	0.282**	–		
4. Intrinsic motivation T2	4.28	0.72	0.355**	0.440**	0.382**	–		4.28	0.80	0.391**	0.503**	0.380**	–	
5. Extrinsic motivation T2	3.07	0.89	−0.024	0.200*	−0.041	0.082	–	3.22	1.02	0.206**	0.161*	0.076	0.248**	–

*p < 0.05; **p < 0.01

Possible differences between experimental and control groups regarding the demographics and the key study variables were also tested. The intervention and control groups did not differ from each other in terms of participants’ gender (*χ*^2^ (1, *N* = 334) = 0.033, *p* = 0.856), age (*t*(332) = −1.92, *p* = 0.056), the number of reciprocal classroom friends (*t*(332) = −0.66, *p* = 0.509), baseline outgroup attitudes (*t*(332) = 0.22, *p* = 0.825), and any of the moderators of the study: T2 levels of friends’ outgroup attitudes (*t*(332) = −0.11, *p* = 0.913), extrinsic motivation (*t*(332) = 1.43, *p* = 0.153), and intrinsic motivation to be non-prejudiced (*t*(332) = −0.03, *p* = 0.487).

As reported in an earlier study utilizing partly the same data (Mäkinen et al., 2022), there was no direct intervention effect on outgroup attitudes: the mean change between pre-intervention (*M* = 74.25, SD = 21.37) and post-intervention (*M* = 73.85, SD = 21.90) scores in the experimental group compared to the mean change between pre-intervention (*M* = 75.90, SD = 21.94) and post-intervention (*M* = 75.15, SD = 22.24) scores in the control group showed no change in attitudes as a result of the intervention (*F*(1,454) = 0.035, *p* = 0.851, *η*^2^ = 0.000). The nonsignificant effect of the intervention on outgroup attitudes in general hold true also for majority (*F*(1,358) = 0.291, *p* = 0.590, *η*^2^ = 0.001) and minority samples (*F*(1,94) = 2.025, *p* = 0.158, *η*^2^ = 0.021) separately.

### Main Analyses

To examine the factors that could explain the effectiveness or ineffectiveness of the intervention for possible sub-groups of participants, the main research questions on whether the effect of the intervention is moderated by peer norms (H1), intrinsic anti-prejudice motivation (H2), or extrinsic anti-prejudice motivation (H3) was tested.

Table 2 shows the results of the hierarchical regression analysis. In Step 1, baseline values of outgroup attitudes, research condition, T1 values of the tested moderators, and majority/minority status as covariates predicted 37 percent (*R*^2^_adj_ = 0.372) of the variance in outgroup attitudes at T2. As expected, outgroup attitudes at T2 were predicted by the baseline values of outgroup attitudes but not by condition, denoting the lack of direct intervention effect. Including outgroup attitudes of reciprocal friends and intrinsic and extrinsic anti-prejudice motivations in Step 2 led to a significant increase in the variance accounted for by the model (*R*^2^_change_ = 0.056; *F*_change_ = 10.81, *p* < 0.001). Only intrinsic anti-prejudice motivation at T2 significantly predicted more positive outgroup attitudes after the intervention.

**Table 2:** Results of the hierarchical linear regression analysis predicting outgroup attitudes after the intervention (*N* = 334)

	Model 1				Model 2				Model 3			
Predictor	*B*	SE *B*	95% CI		*B*	SE *B*	95% CI		*B*	SE *B*	95% CI	
			LL	UL			LL	UL			LL	UL
Intercept	76.03***	1.16	73.75	78.32	75.86***	1.12	73.66	78.05	76.06***	1.11	73.87	78.24
Outgroup Attitudes T1	10.42***	1.09	8.29	12.56	9.50***	1.05	7.43	11.59	9.73***	1.05	7.67	11.80
Condition^a^	−0.99	0.93	−2.81	0.83	−0.73	0.89	−2.47	1.02	−0.65	0.89	−2.39	1.08
Majority/Minority Status^b^	−1.20	1.18	−3.52	1.13	−0.75	1.14	−3.00	1.50	−0.77	1.14	−3.00	1.46
Friends’ Attitudes T1	2.87**	0.97	0.96	4.78	2.14^†^	1.11	−0.03	4.32	2.32*	1.11	0.15	4.50
Intrinsic Motivation T1	2.57*	1.12	0.38	4.49	−0.73	1.24	−3.17	1.72	−0.99	1.26	−3.43	1.44
Extrinsic Motivation T1	1.56	0.95	−0.32	3.39	1.36	1.07	−0.75	3.47	1.41	1.08	−0.69	3.51
Friends’ Attitudes T2					0.16	1.13	−2.06	2.38	0.32	1.15	−1.90	2.54
Intrinsic Motivation T2					6.18***	1.19	3.83	8.52	6.27***	1.21	3.91	8.64
Extrinsic Motivation T2					0.80	1.07	−1.30	2.91	1.33	1.09	−0.80	3.45
Condition × Friends’ Attitudes									1.00	0.97	−0.90	2.90
Condition × Intrinsic Motivation									−0.67	1.00	−2.63	1.30
Condition × Extrinsic Motivation									2.52**	0.93	0.70	4.35
*R*^2^	0.383				0.439				0.453			
*F* change for *R*^2^	33.82***				10.81***				2.72*			

^†^<0.1; *p < 0.05; **p < 0.01; ***p < 0.001

^a^1 = intervention, −1 = control

^b^1 = majority, −1 = minority

Adding the three interaction terms to the model in Step 3 increased explained variance (*R*^2^_change_ = 0.014; *F*_change_ = 2.72, *p* = 0.044). As shown in Table 2, the interaction terms with neither friends’ attitudes nor intrinsic anti-prejudice motivation were predictive of outgroup attitudes after the intervention. Thus, there was no support for Hypotheses 1 and 2. However, regarding Hypothesis 3, there was an interaction effect between condition and extrinsic anti-prejudice motivation on outgroup attitudes. Together, all the predictors in the final model accounted for 43 percent (*R*^2^_adj_ = 0.433) of the variance in post-intervention attitudes, *F*(12, 333) = 22.15, *p* < 0.001).

**Fig. 1:**
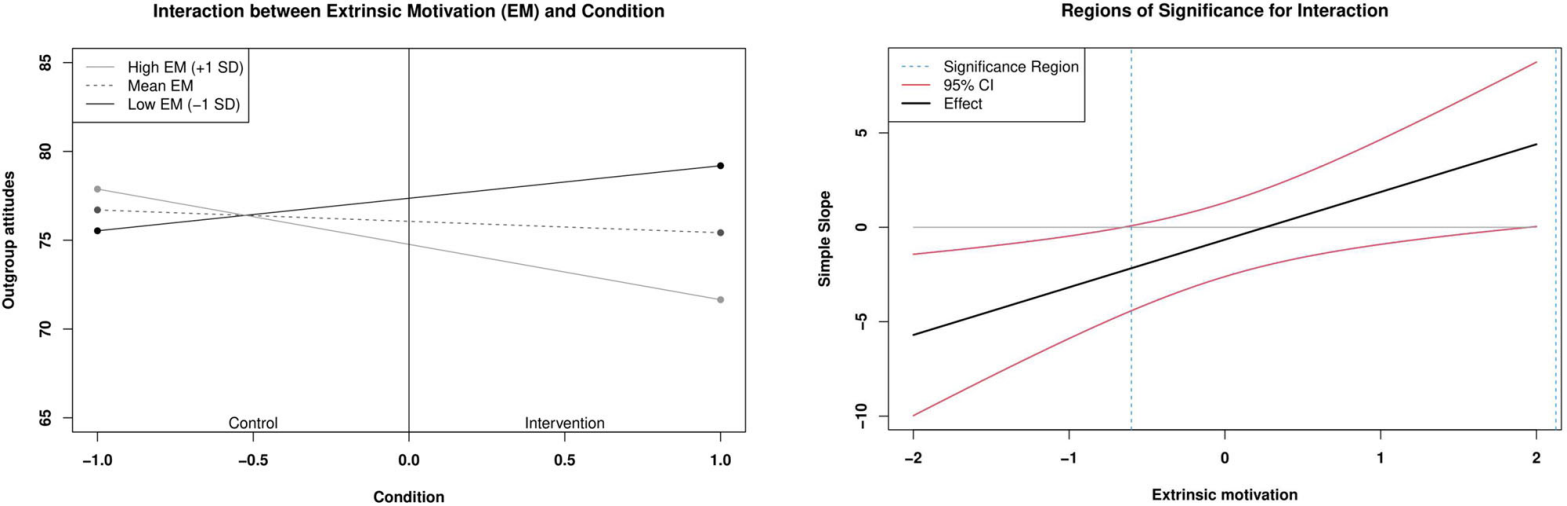
Simple slopes and regions of significance for the interaction between extrinsic motivation and condition predicting outgroup attitudes after the intervention

The interaction between condition and extrinsic anti-prejudice motivation was further explored by probing simple slopes of the intervention effect on outgroup attitudes as a function of the level of extrinsic motivation with 95% confidence bands for regions of significance (Preacher et al, 2006). The results of the analysis of the regions of significance are displayed in Fig. 1. The interaction plot on the left shows that there was a difference in the association of condition and outgroup attitudes among participants with different levels of extrinsic anti-prejudice motivation: among those low in extrinsic motivation, the intervention was associated with less positive outgroup attitudes (*t*(321) = −2.29, *p* = 0.023), while there was no difference in outgroup attitudes between conditions among those high in extrinsic motivation (*t*(321) = 1.31, *p* = 0.192). As shown in the graph on the right, the lower and upper bounds of the region of significance corresponded to extrinsic motivation scores of −0.65 and 1.95, indicating that the simple slope of outgroup attitudes regressed on condition significantly differed from zero for values of extrinsic motivation outside this range. The centered extrinsic motivation ranged from −2.17 to 1.88, meaning that the intervention effect was significant only for relatively low observed values of extrinsic motivation (i.e., scores below −0.65).

### Sensitivity Analysis

As a sensitivity analysis, the regression model was repeated with a measure for friends’ self-reported outgroup attitudes composed of the attitudes of all nominated friends, not only the reciprocal ones. The results remained the same. In the final model (*R*^2^ = 0.45, *F*(3, 351) = 23.49, *p* < 0.01), participants’ outgroup attitudes were predicted by their friends’ pre-intervention attitudes (*β* = 0.12, *t*(351) = 2.46, *p* = 0.014) and intrinsic anti-prejudice motivation at T2 (*β* = 0.37, *t*(351) = 7.08, *p* < 0.01). Regarding the hypothesis testing, only the extrinsic motivation continued to moderate the intervention effect on outgroup attitudes (*β* = 0.08, *t*(351) = 1.99, *p* = 0.048).

## Discussion

In a quest to broaden the understanding of factors that might facilitate or mitigate the efficacy of vicarious contact among adolescents, the present study tested for the role of intrinsic and extrinsic anti-prejudice motivation and self-reported outgroup attitudes of reciprocal friends in the success of a school-based vicarious contact intervention in improving attitudes towards cultural outgroups among Finnish secondary school students. The results showed that adolescents’ intrinsic but not extrinsic anti-prejudice motivation and the pre-intervention attitudes of their reciprocal classroom friends were positively related to adolescents’ outgroup attitudes. However, only extrinsic anti-prejudice motivation was found to moderate the intervention effect. Further examination showed that the intervention had, in fact, a detrimental effect on the students with low extrinsic motivation to be non-prejudiced, while no effect on those students with high extrinsic motivation. This indicates that the vicarious contact effect did not work as expected for students less inclined to conform to anti-prejudice norms to gain social approval.

In this regard, the initial hypothesis on the moderating effect of extrinsic anti-prejudice motivation was not fully accurate. While high extrinsic motivation was expected to facilitate the positive vicarious contact effect on outgroup attitudes, the results indicated low levels of extrinsic motivation to deteriorate the effect of vicarious contact on outgroup attitudes. The rationale for the moderating role of extrinsic anti-prejudice motivation stays nevertheless the same as this result can be interpreted to demonstrate how the message of vicarious contact does not resonate with less extrinsically motivated participants who, unlike highly externally motivated individuals, are not ready to review their perspectives in light of social cues. As vicarious contact operates through establishing and reinforcing positive social norms (Mazziotta et al., 2011), this conclusion aligns with previous studies on how socio-normative cues affect the intentions to respond without intergroup bias among individuals more inclined to these external cues in regulating their behavior (e.g., Burns & Monteith, 2019).

This logic can also be used to explain the lack of support for the hypothesis regarding the intrinsic anti-prejudice motivation facilitating the intervention effect on outgroup attitudes. Highly intrinsically motivated individuals may be better able to self-reflect upon their attitudes and behaviors than extrinsically motivated individuals. This explanation would be in line with the notion that while for externally motivated individuals, significant others constitute the important evaluative audience (i.e., others prescribe the standard), for those whose motivation to respond without prejudice derives from internal standards it is the self and not the external source or norm that serves as the evaluative audience of importance and prescribes the standard for behavior (Plant & Devine, 1998). Furthermore, people who have internalized the anti-prejudice norm as a part of their self-image are also those less in need of prejudice-reduction interventions as intrinsic anti-prejudice motivation is strongly related to positive outgroup attitudes to begin with (e.g., Thijs et al., 2016). This was also demonstrated in this study as intrinsic anti-prejudice motivation was associated with more positive attitudes towards different ethnic and cultural groups regardless of the intervention.

In contrast to intrinsic motivation, extrinsic anti-prejudice motivation is often seen as more harmful to intergroup relations than intrinsic motivation because it generally predicts less positive outgroup attitudes (e.g., Butz & Plant, 2009). Some previous studies have even suggested that attempts to promote positive outgroup attitudes by provoking extrinsic motivation to regulate prejudice can have a counterproductive effect by producing more explicit and implicit prejudice (Legault et al., 2011) and prompting negative affective responses in participants (Plant & Devine, 2001). Thus, ostensibly, there would seem to be a contradiction between the findings of the earlier studies and the results of the current study, indicating that, if anything, high extrinsic motivation was shielding participants from the unintended negative impact of the intervention evident among the students who were less extrinsically motivated to be non-prejudiced. However, it is noteworthy that, unlike these previous experimental studies, the Stories about Friendship intervention did not aim to stimulate extrinsic motivation or compliance to other-imposed prejudice regulation. On the contrary, intervention facilitators were asked to avoid a dictating tone while emphasizing the positive experiences and outcomes of intergroup contact. In case negative experiences and attitudes were expressed during the sessions, the teachers’ manual instructed the facilitators not to suppress or deny students’ negative experiences but to subtly challenge the generalization of their negative views to all outgroup members. As no difference in the levels of extrinsic anti-prejudice motivation between intervention and control groups was detected, it can be assumed that the intervention did not increase students’ extrinsic motivation to be non-prejudiced, which, in the light of previous research, could have been expected to lead to an attitudinal backlash (Plant & Devine, 2001).

Interestingly, the results showed instead a sign of an attitudinal “backlash” among less extrinsically motivated participants taking part in the intervention. Unlike the less extrinsically motivated participants in the control group, their attitudes towards people from different cultural outgroups were less positive at the end of the study. It is possible that among those less interested in complying with social pressure to regulate their prejudice, the social nudging in the form of vicarious contact has not only been ineffective but seen as manipulative and employing too excessive pressure. The defiant backlash, visible as an unanticipated decline in outgroup attitudes, could then be seen as a reaction to this normative control and be explained by a thwarted sense of autonomy, which, according to self-determination theory (Deci & Ryan, 2002), is necessary for eliciting behavioral change. However, to better understand the defiant attitudinal reaction to the intervention, further research would be warranted to examine, for example, how the stories of vicarious contact and the intervention itself are perceived by the participants with different outset of anti-prejudice motivation.

As with the hypothesis on intrinsic anti-prejudice motivation, there was no support for the hypothesis regarding the facilitating role of friends’ attitudes on the effectiveness of the intervention. In other words, friends’ attitudes did neither help nor hinder the efficacy of vicarious contact. The absence of peer influence in explaining the effectiveness of a contact-based intervention bears similarities with the previous findings showing that the perceived peer norms regarding the willingness for intergroup contact did not alter the effectiveness of direct contact intervention in reducing prejudice towards Roma and LGBT minorities among high school students in Hungary (Orosz et al., 2016). However, it is necessary to acknowledge that peer influence has also proven important for successfully implementing school-based interventions utilizing indirect contact methods such as imagined contact (Smith & Minescu, 2022) and vicarious contact (Cocco et al., 2022). However, in contrast to the present study that examined peers’ self-reported attitudes, these previous studies incorporated peer norms as a part of the intervention design, i.e., experimentally manipulating the presence of peer influence by including cooperation with peers (Cocco et al., 2022) or by asking the participants to imagine the presence of ingroup peers in an imagined contact setting with an outgroup member (Smith & Minescu, 2022). This might suggest that friends and peers are crucial in advancing the effect of prejudice-reduction interventions, but their effect in boosting the intervention is not as strong when not seen “in action”. This could be due to people not necessarily having an accurate perception of the attitudes and values of others, even of their close partners or family members, instead projecting their own values and attitudes on them (Stattin & Kim, 2018). Furthermore, from the social learning perspective, being aware of the existence of a particular social norm would not be enough to elicit adherence to it unless others are believed to do so as well (Gross & Vostroknutov, 2022). It is possible that the attitudes held by classroom friends were not sufficiently communicated or linked to the intervention and, thus, not salient enough for the participants of this study to impact the effectiveness of the intervention.

Reflecting on the role of peer norms in the effectiveness of prejudice-reduction intervention among youth, it is also necessary to consider the developmental aspects. As mentioned earlier, findings from recent longitudinal studies have shown the impact of peer norms starting to decline in late adolescence (see e.g., Ahmed et al., 2020). It has, for example, been suggested that the increasing resistance to peer influence would occur between the ages of 14 and 18 (Steinberg & Monahan, 2007). The participants in this study represented adolescents aged 13 to 15 who were perhaps approaching or had just reached this transitional stage at the time of the study. This contrasts the current study with the previous studies indicating a positive effect of peer norms on the prejudice-reduction intervention among primary school students in Italy (Cocco et al., 2022) and Ireland (Smith & Minescu, 2022). The developmental stage of the participants may thus partly explain why the friends’ attitudes did not affect the intervention effect on outgroup attitudes in this study.

When interpreting the results of the present study, it is also important to address some of its limitations. Firstly, the number of participants was reduced considerably due to the high drop-out rate and the missing data on friend nominations. As it would not have been possible to reliably replace the missing nominations of participants’ classroom friends, the missing data was handled with listwise deletion. Because of the missingness, the proportion of nonparticipating students from each class would have been too high for conducting social network analysis (Huisman & Steglich, 2008), which otherwise could have offered a more nuanced view of the peer influence inside the classrooms studied. For example, future studies might want to examine the most potent norm agents within classrooms, whose influence might be crucial in facilitating attitude change in the rest. Secondly, as also related to the friendship nominations, it is important to note the possible bias in the data due to the defect in the questionnaire settings, which led to a number of participants being able to nominate more friends than instructed. However, the analysis was restricted only to the attitudes of reciprocal friends, which can be considered to some extent diminish the bias in the discrepancy of the number of nominated friends between participants.

## Conclusion

Vicarious contact has often been used in intervention studies aiming to reduce prejudice among youth in educational settings. However, as these interventions may also prove ineffective, there is a dire need for examining relevant determinants of the effectiveness of such interventions while also considering how the maturational developments in social-affective processing during adolescence might play a role in what determinants to target. The present study focused on social influence as one such determinant by testing the moderating effect of friends’ attitudes and the levels of internally/socially derived anti-prejudice motivation. The results showed that adolescents’ intrinsic, but not extrinsic, anti-prejudice motivation and the pre-intervention attitudes of their reciprocal classroom friends predicted positive attitudes towards people from different ethnic and cultural groups. However, only extrinsic anti-prejudice motivation was found to moderate the intervention effect, indicating that the intervention had a detrimental effect on outgroup attitudes among adolescents less motivated to avoid being prejudiced for the reason of social acceptance. To conclude, the results of this study drew critical attention to the limits of the effectiveness of vicarious contact as a tool to reduce prejudice in schools. The study further suggests that in attempts to reduce prejudice among adolescents, especially through means involving normative influence as a mechanism of change, motivational aspects in regulating prejudice should not be overlooked, as the results pointed toward a potential attitudinal backlash effect in tackling ethnic prejudice among adolescents less susceptible to the social influence of others.

## Appendix

An example of a friendship story used in the intervention (translated from Finnish).

*Ville is a seventh grader whose favorite school subject is P.E. He has soccer practice three times a week*.

“Before, I didn’t really know what to think of foreigners, although some attended our school. It felt hard to do group work or something like that together with them. I just didn’t know what to do or talk about with them. I wondered if I could hang out with them like with Finns. Thinking back on it now, it was an unnecessary fear. Then some of my friends started hanging out with Hakim, who was new to our soccer team, and said he’s a great guy. I eventually got to know him through my friends. I realized that he’s interested in the same things as me, even though he has some different ways of doing things.

We usually meet up with Hakim at practice and sometimes at other times, too. He’s better than me at soccer, and I’ve learned all these new tricks from him. Sometimes, I think he’s a hundred times more easy-going than some of my Finnish friends. Once, I saw how some Finnish men started shouting insults at him outside when he spoke Arabic on the phone. I started thinking about how it would feel to be bashed just because I’m not from here. What if I had to move to a different country and nobody wanted to be friends with me because I’m Finnish? I probably think about these things more now than I used to.”
